# Photothermal ablation of murine melanomas by Fe_3_O_4_ nanoparticle clusters

**DOI:** 10.3762/bjnano.13.20

**Published:** 2022-02-22

**Authors:** Xue Wang, Lili Xuan, Ying Pan

**Affiliations:** 1 Department of Obstetrics & Gynecology, China–Japan Union Hospital of Jilin University, Changchun, People’s Republic of China

**Keywords:** Fe_3_O_4_ nanoparticle clusters, heat shock protein 70, melanoma, near infrared, photothermal therapy

## Abstract

Melanoma is one of the deadliest forms of cancer, for which therapeutic regimens are usually limited by the development of resistance. Here, we fabricated Fe_3_O_4_ nanoparticle clusters (NPCs), which have drawn widespread attention, and investigated their role in the treatment of melanoma by photothermal therapy (PTT). Scanning electron microscopy imaging shows that our synthesized NPCs are spherical with an average diameter of 329.2 nm. They are highly absorptive at the near-infrared wavelength of 808 nm and efficient at locally converting light into heat. In vitro experiments using light-field microscopy and cell viability assay showed that Fe_3_O_4_ NPCs, in conjunction with near-infrared irradiation, effectively ablated A375 melanoma cells by inducing overt apoptosis. Consistently, in vivo studies using BALB/c mice found that intratumoral administration of Fe_3_O_4_ NPCs and concomitant in situ exposure to near-infrared light significantly inhibited the growth of implanted tumor xenografts. Finally, we revealed, by experimental approaches including semi-quantitative PCR, western blot and immunohistochemistry, the heat shock protein HSP70 to be upregulated in response to PTT, suggesting this chaperone protein could be a plausible underlying mechanism for the observed therapeutic outcome. Altogether, our results highlight the promise of Fe_3_O_4_ NPCs as a new PTT option to treat melanoma.

## Introduction

The global incidence of melanoma, one of the deadliest forms of cancer, has kept increasing annually over the past decades at an accelerating rate [1]. Depending on the features of melanomas, therapeutic options include surgical resection, chemotherapy, immunotherapy, photodynamic therapy and several others. Although these treatments could initially meet therapeutic needs, their efficacies commonly drop afterwards due to adverse effects or development of various resistance mechanisms, making the advent of novel strategies imperative for early diagnosis and efficient treatment [2].

Photothermal therapy (PTT) is a recently developed regimen that requires administration of nanomaterials with unique optical properties to absorb and locally convert near-infrared (NIR) light into heat [3]. Nanoscale agents tend to accumulate within tumor sites due to the enhanced permeability and retention (EPR) effect. Also, tumor cells are more sensitive to elevated temperatures than normal cells. Thus, PTT specifically ablates tumor cells, while leaving healthy neighbor tissues intact [4–5]. Treatment specificity is further guaranteed by the high transmissivity and low absorption of NIR light when penetrating through biological tissues, making PTT a minimally invasive approach for tumor intervention.

One such type of nanomaterial that has received considerable attention is Fe_3_O_4_ core-based nanoparticles, which have been approved by the Food and Drug Administration (FDA) as safe biomaterial with no long-term toxicity [6–7]. The superparamagnetic properties make them ideally suited for many biomedical applications, such as MRI imaging, targeted drug delivery and hyperthermia therapy [8–9]. Hyperthermia therapy can be achieved by using either magnetic fields or NIR irradiation. Application of an external alternating magnetic field on these nanoparticles leads to the production of heat to mediate magnetic hyperthermia, whereas exposure to and subsequent absorption of NIR light by iron oxide nanoparticles promotes NIR-induced hyperthermia [10]. Although magnetic hyperthermia has been widely used in biomedical research, it is subject to several limitations such as the need for sophisticated equipment, cellular confinement and lower hyperthermia efficiency [11]. Indeed, other researchers find that the temperature increase by magnetic hyperthermia is much lower than that of NIR-induced heating, presumably due to the coating layers needed for biological dispersion [12]. Yu et al. first discovered strong photothermal effects of alumina-coated Fe_3_O_4_ nanoparticles against bacteria upon exposure to NIR light [13]. Further studies with esophageal cancer demonstrate that Fe_3_O_4_ nanoparticles induce hyperthermia post absorption to suppress tumor growth in a dosage-dependent manner [14]. However, due to very high dosage needed to elicit sufficient hyperthermia by NIR irradiation, lingering magnetite may impose potential systemic toxicity. Thus, Fe_3_O_4_ single nanoparticles must be modified to reduce the dosage while keeping their therapeutic efficacy.

In our earlier report, we synthesized Fe_3_O_4_ nanoparticle-containing nanoprobes, which were doped with lanthanide elements to allow for up-conversion luminescence imaging [15]. We assessed their photothermal effects in both A375 cells and BALB/c mice models. However, the synthesis of these nanoprobes requires complex procedures and is economically challenging. Recent findings show that clustered magnetic Fe_3_O_4_ nanoparticles induce a redshift in the light absorption spectra, which enhances light absorbance within the NIR region and improves their utilization as photosensitizers during PTT to ablate lung tumors [16]. However, the role of Fe_3_O_4_ nanoparticle clusters in the treatment of cutaneous melanoma has remained unknown.

In this study, we synthesized Fe_3_O_4_ superparamagnetic nanoparticle clusters, examined their morphology by scanning electron microscopy (SEM) and tested their capacity of light-to-heat conversion. Then, we evaluated the effectiveness of the as-synthesized nanoclusters as PTT agents both in vitro and in vivo, and finally explored the putative molecular mechanisms underlying the observed therapeutic effects.

## Results and Discussion

### Properties of Fe_3_O_4_ nanoparticle clusters (NPCs)

**Figure 1:**
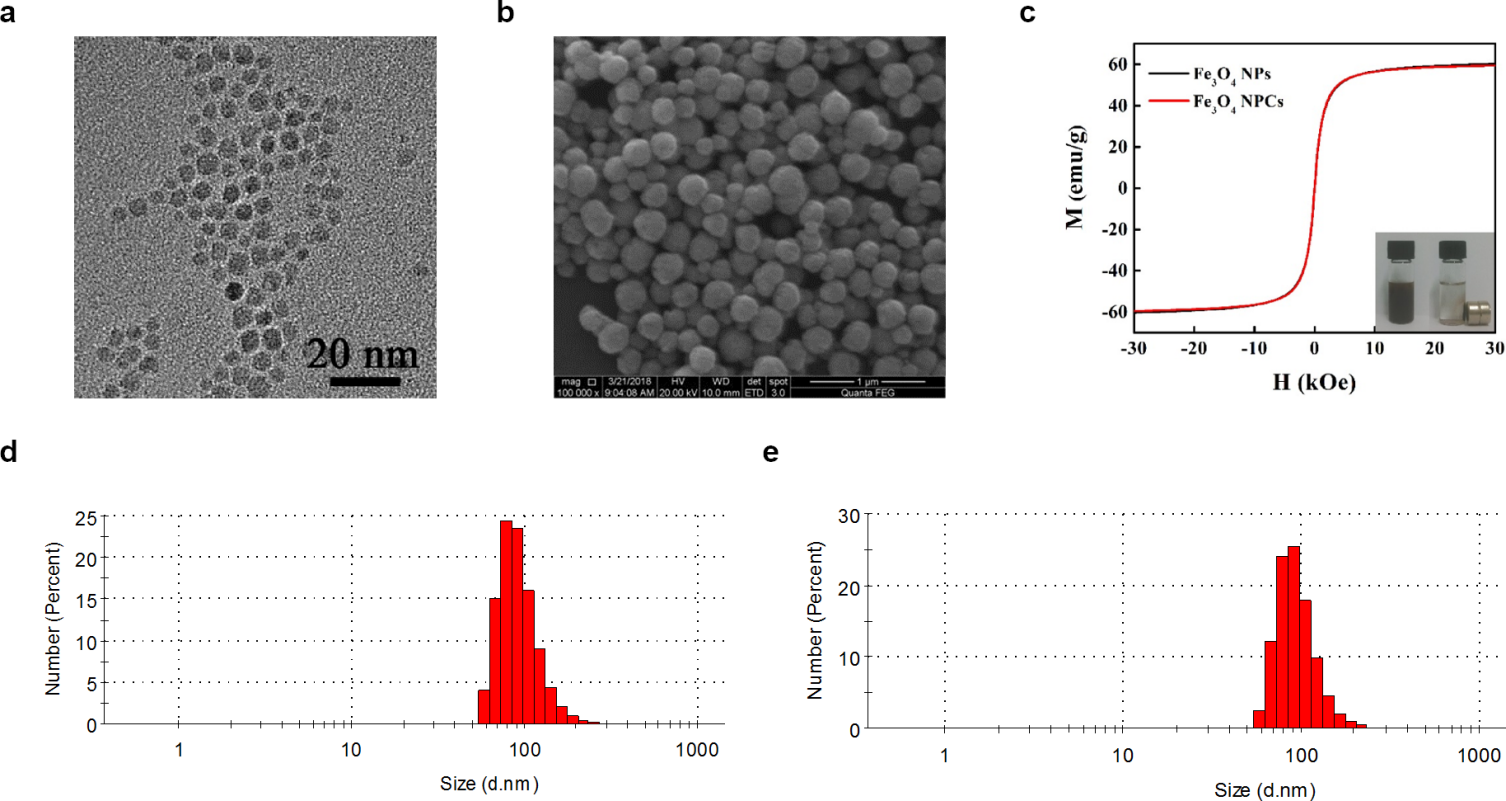
Characterization of the superparamagnetic Fe_3_O_4_ nanoparticle clusters. (a) TEM image revealing cubic morphology of individual Fe_3_O_4_ nanoparticles with a uniform diameter of ca. 5.2 nm. Scale bar: 20 nm. (b) SEM image of clustered Fe_3_O_4_ nanoparticles with an average diameter of 329.2 nm. Scale bar: 1 µm. (c) Magnetic hysteresis curves of individual Fe_3_O_4_ nanoparticles as well as NPCs. The insert shows aggregation of NPCs dispersed in aqueous solution by a magnet. (d) DLS characterization of NPCs dispersed in cell culture medium. (e) DLS results of NPCs suspension after five heating/cooling cycles.

The as-synthesized individual Fe_3_O_4_ nanoparticles were evenly dispersed in chloroform solution with high thermal stability. Transmission electron microscopy (TEM) revealed a typical face-centered cubic structure with uniform size and an average diameter of 5.2 ± 1.5 nm (Figure 1a) [17]. After the surfactant dodecyltrimethylammonium bromide (DTAB) was introduced, its lipophilic ends combined with the oleic acid and oleylamine ligands present on the surface of Fe_3_O_4_ nanoparticles through van der Waals forces to facilitate the dispersion of nanoparticles in aqueous solution. Further addition of ethylene glycol weakened the van der Waals interaction, causing decomposition of nanoparticle micelles and the formation of Fe_3_O_4_ nanoparticle clusters, which were then coated with polyvinylpyrrolidone (PVP) to improve stability, biocompatibility and chemical features of the nanoparticles. Further SEM measurements revealed the as-formed Fe_3_O_4_ NPCs to be spherical and uniformly sized with an average dimension of 329.2 nm (Figure 1b). Moreover, SEM imaging at higher magnification showed that the clusters were composed of many regularly organized small nanoparticles, preventing the loss of superparamagnetism due to increased particle size. These NPCs possess high magnetization with a saturation value of 59.4 emu/g (Figure 1c) and stronger absorption intensities at the NIR wavelength of 808 nm than individual nanoparticles [17]. In addition, the dynamic light scattering (DLS) analysis of NPCs suspended in aqueous culture medium reflected good dispersion (Figure 1d). The colloidal stability of our synthesized Fe_3_O_4_ NPCs was confirmed by the conserved DLS pattern of size distribution after five heating/cooling cycles with a NIR laser (Figure 1e). Altogether, our data confirmed the successful fabrication of superparamagnetic Fe_3_O_4_ NPCs that are spherical, uniformly sized and highly absorptive in the NIR region, validating their potential use as PTT agents.

### Photothermal effect of Fe_3_O_4_ NPCs

**Figure 2:**
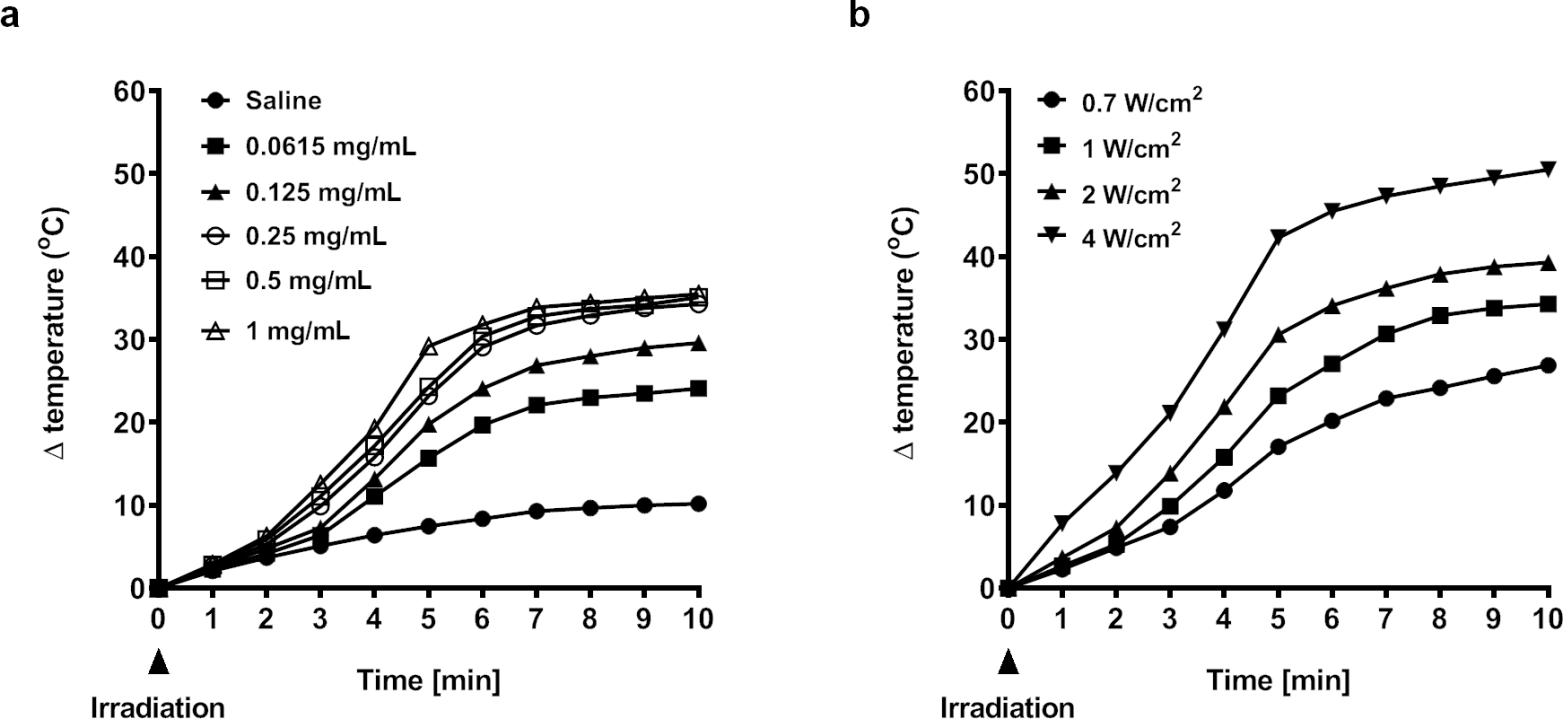
Photothermal conversion of Fe_3_O_4_ nanoparticle clusters. (a) Temperature elevation of aqueous solutions with increasing concentrations of NPCs in response to 808 nm laser irradiation at power density of 1 W·cm^−2^. (b) Temperature elevation of solutions with 0.25 mg/mL NPCs in response to 808 nm laser irradiation at increasing power densities.

To assess their photothermal effect, we measured the increments of temperature at different concentrations of Fe_3_O_4_ NPCs during 10 min of irradiation with an 808 nm laser in vitro. In general, the Fe_3_O_4_ NPC-induced photothermal effect was dosage-dependent, because NPCs at higher concentrations performed markedly better than at lower ones (Figure 2a). Importantly, at concentrations as low as 0.0615 mg/mL, Fe_3_O_4_ NPCs rapidly and significantly raised the solution temperature after NIR irradiation in only 1 min at a power density of 1 W·cm^−2^. This effect was time-dependent with longer irradiation leading to higher temperatures. Eventually, the solution temperature was elevated at the end of irradiation by a net increment of 24.1 °C. In contrast, only a modest increase of 10.2 °C was observed for saline solution, validating the photothermal effect was mainly attributed to Fe_3_O_4_ NPCs. As expected, stronger power densities led to markedly higher temperatures (Figure 2b). Altogether, our results support a rapid and strong photothermal conversion capacity of Fe_3_O_4_ NPCs that carry beneficial potential as photothermal agents.

### Photothermal ablation of tumor cells in vitro

**Figure 3:**
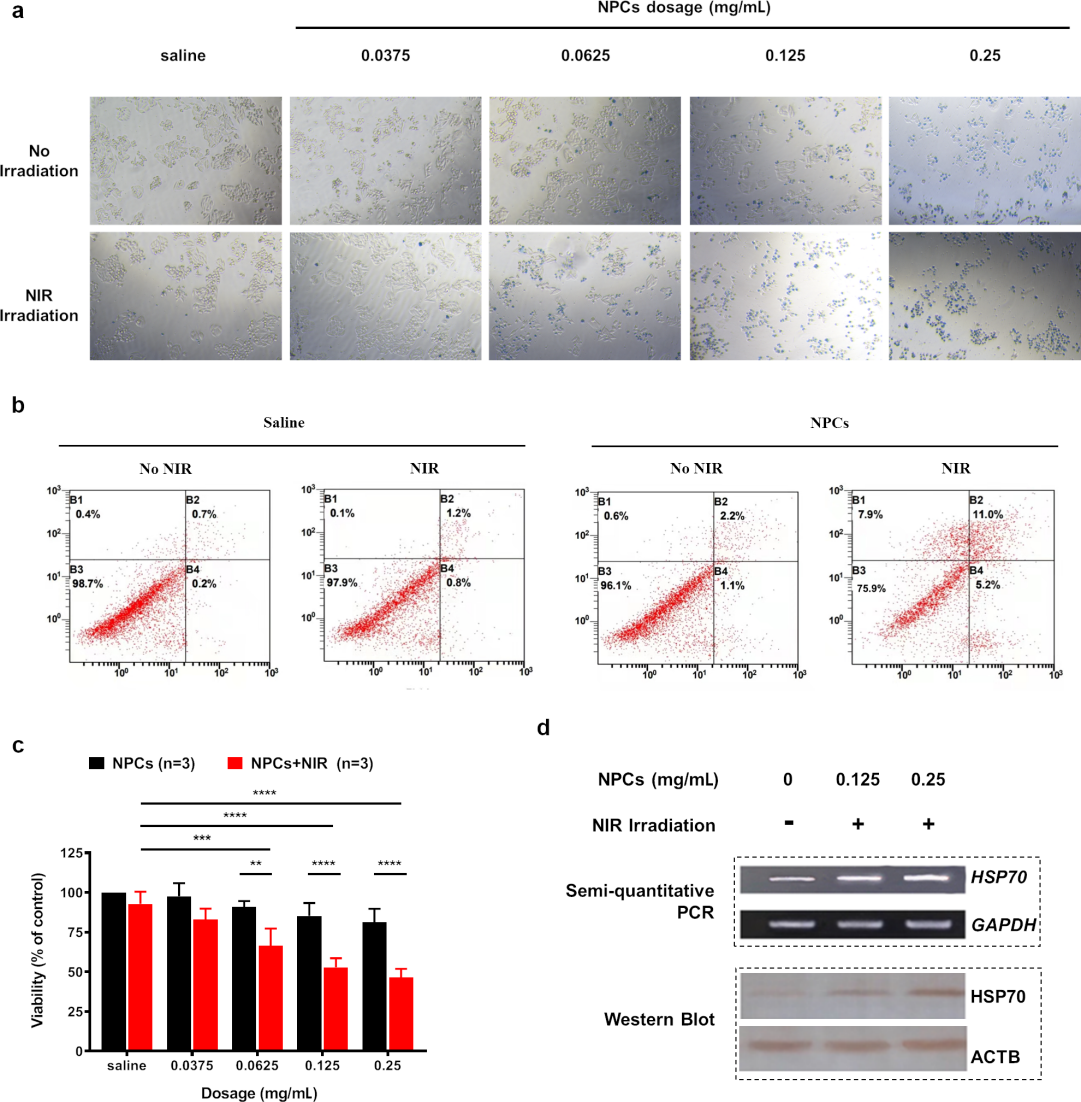
In vitro photothermal ablation of A375 cells. (a) Bright-field microscopy showing the health conditions of A375 cells treated with saline or increasing concentrations of NPCs, in the presence or absence of NIR irradiation. Cells were stained with Trypan blue dye to visualize dead cells. (b) Apoptotic and necrotic rates of A375 cells, as revealed by flow cytometry, after photothermal treatment with 0.0625 mg/ml NPCs under 10 min of NIR irradiation. (c) MTT assay showing viability of A375 cells after their incubation with NPCs at different concentrations with or without NIR exposure. ^**^p < 0.01, ^***^p < 0.001, ^****^p < 0.0001 calculated by two-way ANOVA adjusted by Sidak’s multiple comparisons. (d) Expression of HSP70 in A375 cells was increased after treatment with NPCs (0.125 and 0.25 mg/mL) under NIR irradiation. This increase was observed on both mRNA and protein levels as revealed by semi-quantitative PCR and western blot, respectively.

The dissolution behavior of our finely dispersed Fe_3_O_4_ NPCs was confirmed by DLS results (Figure 1d,e). Furthermore, the successful uptake of Fe_3_O_4_-based nanoparticles by A375 cells has been verified in our previous report [4]. Thus, to test the therapeutic effect of the NPCs in vitro, we first incubated A375 cells with increasing concentrations of Fe_3_O_4_ NPCs (0.0375, 0.0625, 0.125, 0.25 mg/mL) or saline control, then treated them with NIR irradiation for 10 min or did not irradiate them at all. After 24 h, we stained the cells with trypan blue and found that during NIR irradiation NPCs caused overt apoptosis and necrosis in a dosage-dependent manner (Figure 3a). The strongest effect was observed for the sample with 0.25 mg/mL NPCs. NPCs alone, on the contrary, did not affect cell viability at low concentrations and only caused signs of mild cellular toxicity at high concentrations. Similarly, cells in the saline + NIR sample seemed as healthy as those in the saline control group. Consistent with these microscopy observations, flow cytometry analysis, which utilized Annexin V and propidium iodide to quantitate apoptosis and necrosis, showed that the percentage of apoptotic/necrotic cells increased from 1.3% in the saline control group to 24.1% in cells treated with 0.0625 mg/mL NPCs plus NIR irradiation (Figure 3b). Despite the MTT (3-(4,5-dimethylthiazol-2-yl)-2,5-diphenyltetrazolium bromide) assay is prone to multiple interferences, including metal-based nanoparticles, this method has long been regarded as the gold standard for cell viability and proliferation studies, and thus been applied extensively in studies of metal-containing nanoparticles [18–20]. In accordance with our flow cytometry findings, the MTT viability assay showed that in combination with NIR irradiation, Fe_3_O_4_ NPCs led to significant cell death in a dosage-dependent manner, with the efficacious concentrations starting at as low as 0.0625 mg/mL (Figure 3c). The strongest effect was observed for 0.25 mg/mL NPCs, which, when compared to saline-alone control, reduced cell viability to 39.3%. NPCs alone tended to decrease cell viability at higher dosages but did not reach statistical significance, whereas cells exposed to saline + NIR remained unchanged. In this sense, the PTT efficiency of the current NPCs was greater than that of our previously synthesized Fe_3_O_4_ nanoparticle-containing up-conversion nanoprobes, which required a minimal working concentration of at least 0.25 mg/mL to achieve a similar cell viability reduction [15]. These findings indicate that our Fe_3_O_4_ NPCs possess high biocompatibility and are able to ablate melanoma cells at low concentrations under NIR irradiation.

HSP70 is frequently activated in response to heat stress, and earlier studies have found that it promotes apoptosis at least by eliciting antitumor immune responses [21]. This chaperone protein has been shown in our earlier study to be involved in mediating the photothermal effects of Fe_3_O_4_ nanoparticle-containing up-conversion nanoprobes [15]. To check whether HSP70 was involved in Fe_3_O_4_ NPC-mediated PTT, we treated A375 cells for 24 h with NPCs at concentrations of 0.125 or 0.25 mg/mL, followed by 808 nm laser irradiation. Naïve cells were incubated with saline and served as normal controls. Then, we measured the HSP70 expression by semi-quantitative RT-PCR and western blot. We found that the expression of HSP70 was markedly increased on both mRNA and protein levels (Figure 3d), suggesting a critical role of it in mediating the observed therapeutic outcomes. Interestingly, upregulation of HSP70 was specific to NPC-induced hyperthermia, because the HSP70 mRNA level was unchanged after NIR irradiation alone (Supporting Information File 1, Figure S1), albeit NIR itself elevated the solution temperature by 10 °C (Figure 2a).

### Photothermal ablation of tumor cells in vivo

**Figure 4:**
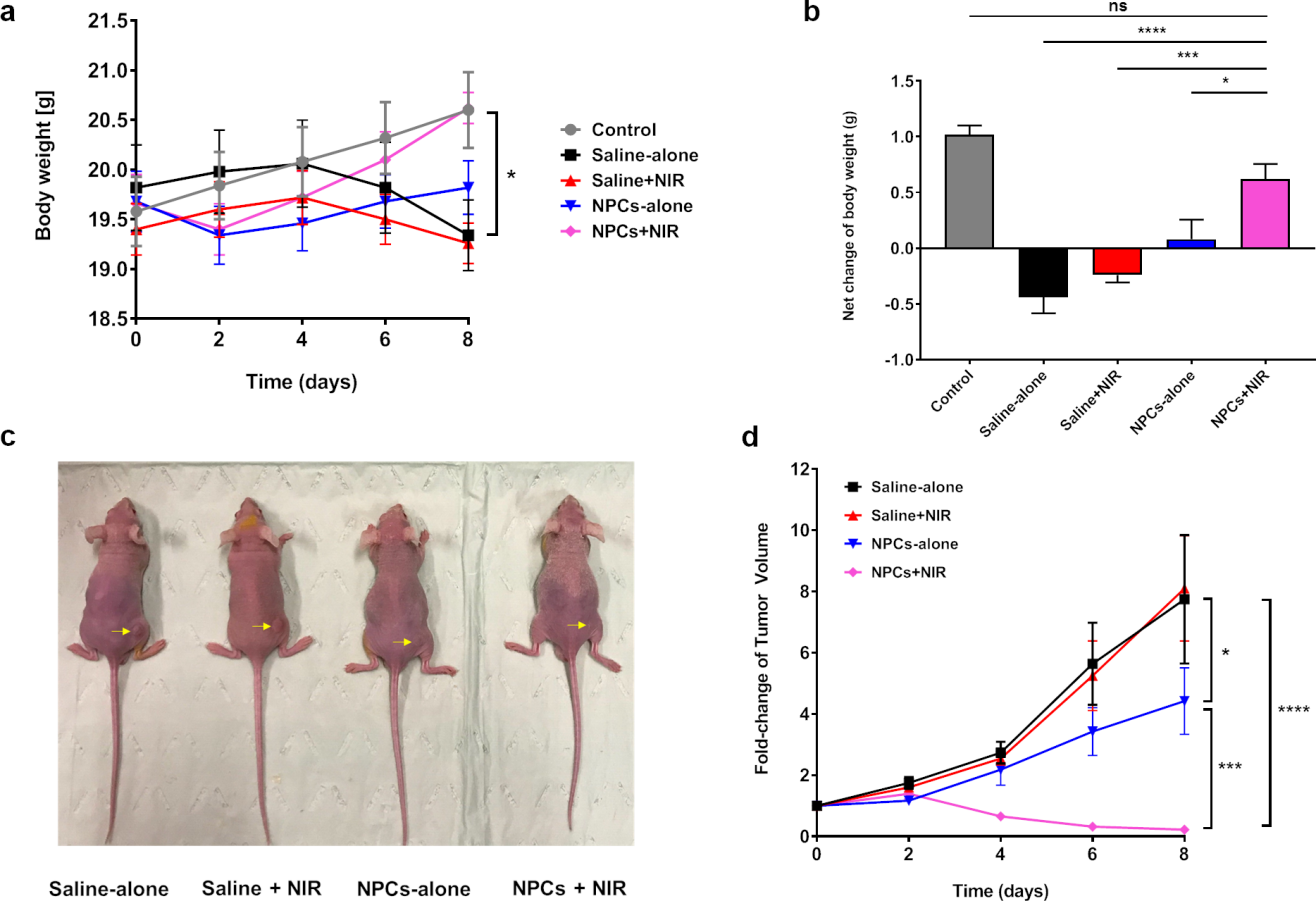
In vivo photothermal therapy in BALB/c mice bearing tumor xenografts. (a) Changes of body weight over the 8-day period post PTT. ^*^p < 0.05, control vs NPCs + NIR at day-8, calculated by two-way repeated measures ANOVA adjusted by Tukey’s multiple comparisons, *n* = 8. (b) Net body weight changes by the end of the study (day 8 versus day 0), ^*^p < 0.05, ^***^p < 0.001, ^****^p < 0.0001 calculated by one-way ANOVA adjusted by Dunnett’s multiple comparisons, *n* = 8. (c) Representative photographs of tumor-bearing mice in each treatment group by the end of the study. Yellow arrows indicate tumor location. (d) Growth curves of tumors in each treatment group. Tumor size was first normalized to its initial size (day-0), then expressed as fold-change over the latter. ^*^p < 0.05, saline-alone vs NPC-alone; ^***^p < 0.001, NPC-alone vs NPCs + NIR; ^****^p < 0.0001, saline-alone vs NPCs + NIR; two-way repeated measures ANOVA adjusted by Tukey’s multiple comparisons, *n* = 8.

To test their therapeutic efficacies in vivo, we first implanted A375 cells subcutaneously into BALB/c mice to form solid tumors. Then, the mice randomly assigned into the five treatment groups specified in the Experimental session. Briefly, these tumor-bearing mice were injected intratumorally with either Fe_3_O_4_ NPCs (2.5 mg/kg) or saline vehicle control and then either exposed or not to NIR irradiation. Undisturbed BALB/c mice were maintained intact and served as normal control. Body weights, tumor volumes, and general health conditions were monitored and recorded every other day over eight days. Our results found that the body weight of the normal control BALB/c mice rose steadily with a net final increment of 1.02 g (Figure 4a,b). In contrast, the saline-alone group showed reduced body weight accompanied by a rapid growth of tumors that increased 7.75-fold after eight days (Figure 4c,d). Neither body weight nor tumor size was significantly different between saline + NIR and saline-alone groups, suggesting a negligible effect of NIR irradiation on biological tissues. Noticeably, we observed an initial drop of body weight in the NPC-alone group during the first four days (Figure 4a), which implied our synthesized NPCs might possess light toxicity, a phenomenon echoed by our light microscopy findings (Figure 3a). Accordingly, tumor volumes of this group were smaller than those of the saline-alone group (Figure 4c,d). The quick restoration of body weight post day 4 demonstrated that these materials are well tolerated and can be cleared out efficiently. Of importance, the combination of NPCs with NIR irradiation dramatically shrank the tumors and reduced their volumes by 77.8% (Figure 4c,d). As a result, the body weights of these photothermally treated mice completely recovered after an initial drop on day 2 (Figure 4a), confirming the applicability of Fe_3_O_4_ NPCs as effective PTT agents. Similar to in vitro findings, the PTT efficiency of the current NPCs in vivo was greater than that of our previously synthesized Fe_3_O_4_ nanoparticle-containing up-conversion nanoprobes, which was administered to mice at a higher dosage of 5 mg/kg [15]. The extent of tumor volume shrinkage is not different between these two studies. Finally, no abnormalities in general health status, defecation, or urination were seen for all mice (data not shown).

**Figure 5:**
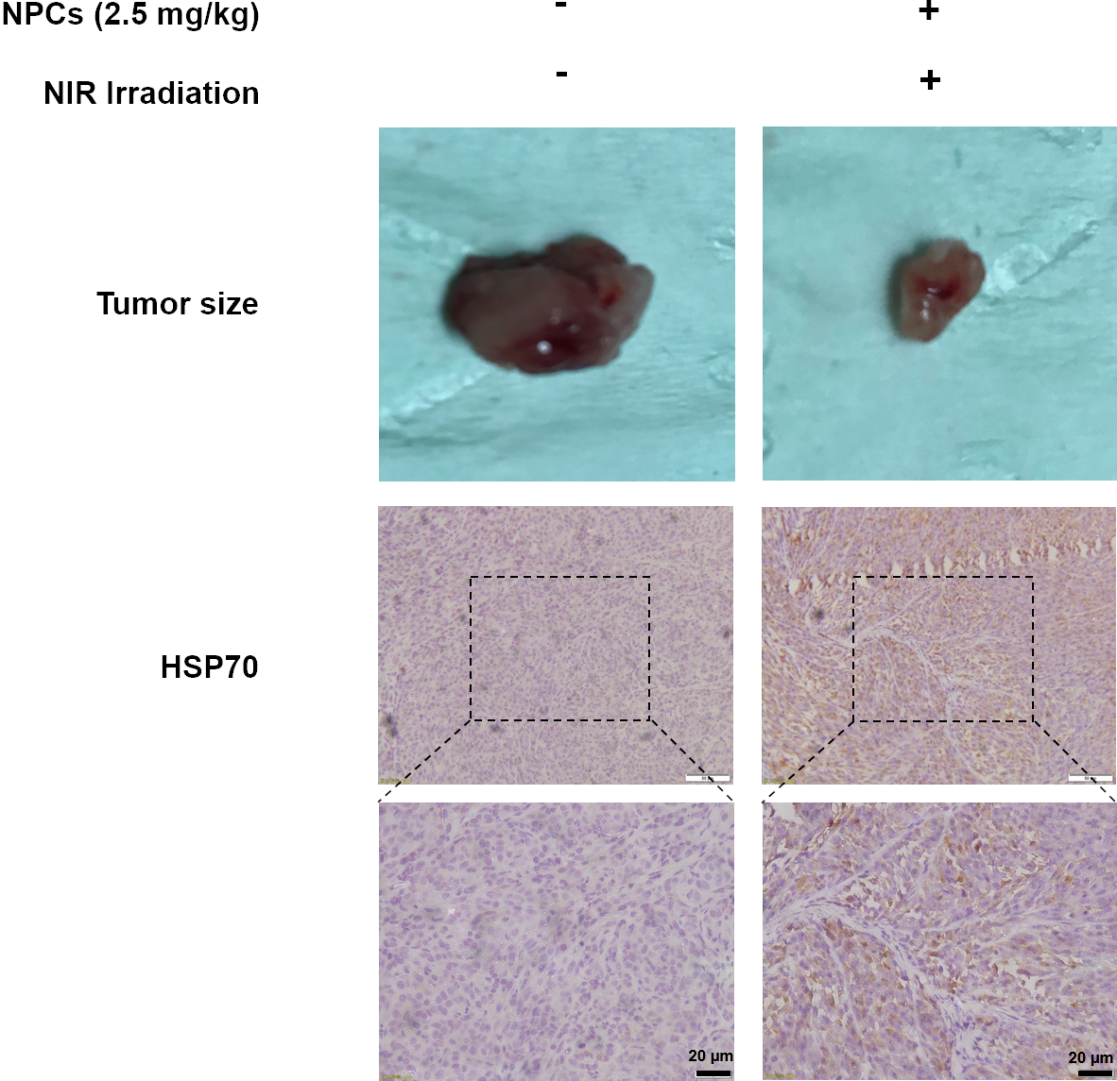
Photothermal therapy increases tumoral level of HSP70. Tumors were isolated from mice that received intratumor injection of either saline without NIR exposure or NPCs (2.5 mg/kg) followed by NIR irradiation. Immunohistochemical staining of harvested tumors revealed HSP70 to be elevated (brown signals) by PTT.

To explore the molecular mechanisms underlying PTT, we performed immunohistochemistry to check the tumoral HSP70 expression in saline-alone and NPCs + NIR groups. Similar to in vitro findings, the reduced tumor size was accompanied by markedly upregulation of HSP70 (Figure 5; Supporting Information File 1, Figure S2), confirming the involvement of HSP70 in Fe_3_O_4_ NPC-mediated PTT in vivo.

## Conclusion

**Figure 6:**
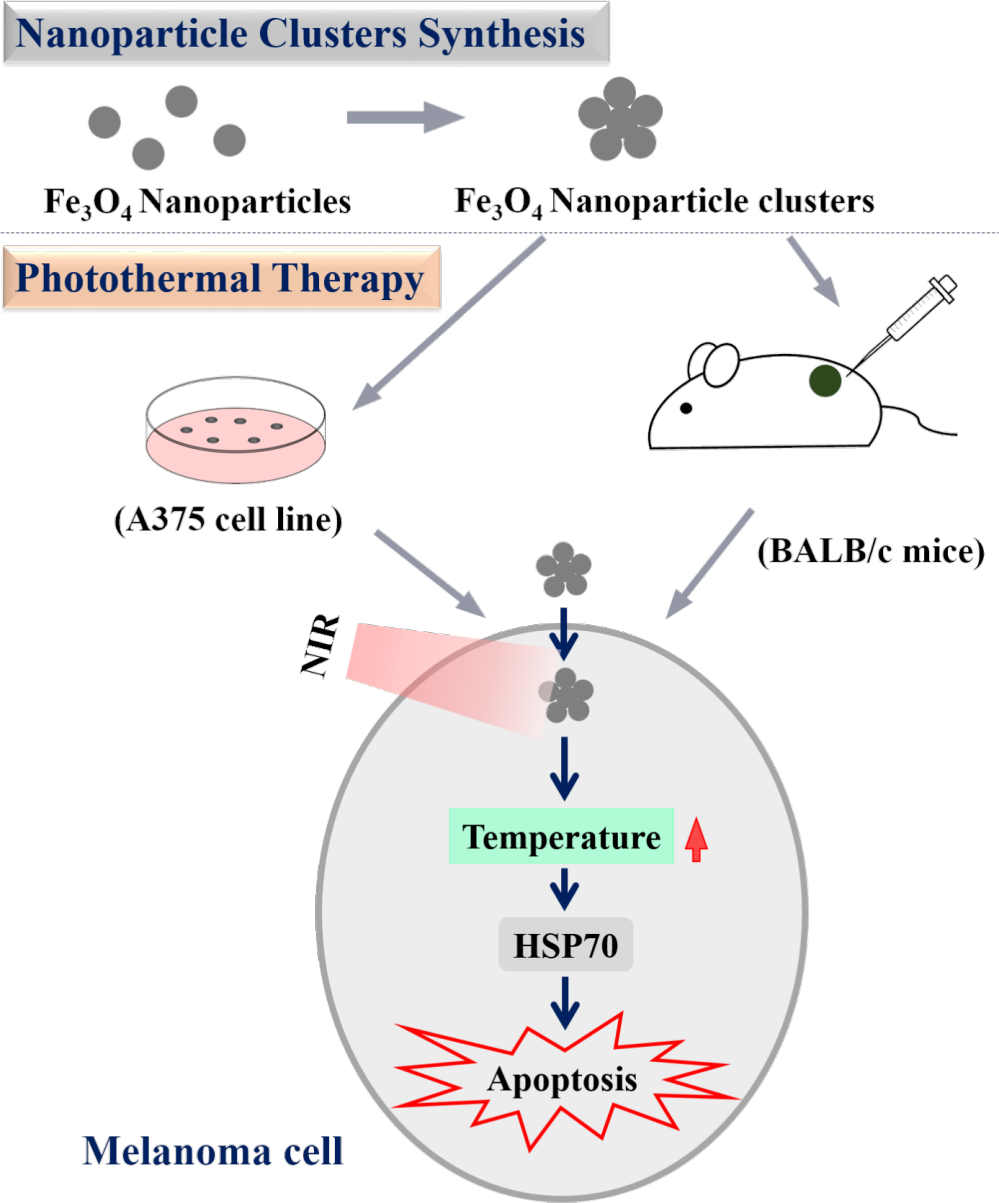
Schematic representation of Fe_3_O_4_ NPC-mediated photothermal therapy in melanoma.

In summary (Figure 6), we fabricated Fe_3_O_4_ nanoparticle clusters of uniform spherical shape, with high absorption at the near-infrared 808 nm wavelength, superparamagnetism, and a strong capacity of photothermal conversion. Both in vitro studies using an immortalized A375 melanoma cell line and in vivo research using xenografted BALB/c mice model confirmed these nanoclusters, under NIR irradiation, led to overt cellular apoptosis and halted growth of implanted tumor xenografts at concentrations that did not elicit cytotoxicity when administered alone. Mechanistically, we discovered the heat shock protein HSP70 as a plausible explanation for the observed therapeutic benefits as a result of hyperthermia. Findings of the current study accentuate the potential application of Fe_3_O_4_ nanoparticle clusters in the treatment of melanoma.

## Experimental

### Reagents and animals

Iron acetylacetonate (Fe(acac)_3_, 99.9+%), dibenzyl ether (99%), oleic acid (90%), oleylamine (>70%), dodecyltrimethylammonium bromide (DTAB, 99%), tetradecyltrimethylammonium bromide (TTAB, 99%), and polyvinylpyrrolidone (PVP, molecular weight 58,000) were purchased from Sigma-Aldrich. Decayltrimethylammonium bromide (DeTAB, 99%) and 1,2-hexadecanediol (97%) were purchased from TCI Chemical Industry Development Co., Ltd. (Shanghai). Chloroform (99%), ethylene glycol (96%), and ethanol (99.7%) were purchased from Beijing Chemical Works. Nitrogen gas was purchased from Juyang Gas (Changchun). Hela cells were purchased from the Cell Bank at the Chinese Academy of Sciences (Shanghai, China). MTT (3-(4,5-dimethylthiazol-2-yl)-2,5-diphenyltetrazolium bromide), penicillin/streptomycin and other biological reagents were purchased from Sigma-Aldrich (St. Louis MO, USA). The BALB/c mice (7–8 weeks old) were provided by the Comparative Medicine Center of Yangzhou University. All animal experiments conducted in the current study were approved by the Institutional Animal Care and Use Committee (IACUC) of Jilin University (including guidelines for animal care, use, and euthanasia).

### Synthesis of individual Fe_3_O_4_ nanoparticles

The Fe_3_O_4_ nanoparticles were synthesized via the thermal decomposition of precursor method as previously reported [22]. Specifically, under nitrogen atmosphere, a solution containing 0.01 mol 1,2-hexadecanediol and 20 mL dibenzyl ether was magnetically stirred within a three-necked flask (100 mL). Then, 0.006 mol oleic acid, 0.006 mol oleylamine, and 0.002 mol Fe(acac)_3_ were added sequentially into that flask to mix under continued stirring for 15 min. Next, the as-prepared mixture was gradually heated up to 200 °C at a rate of 20 °C/min and was kept under stirring for another hour at this temperature. Thereafter, the abovementioned mixture was heated to and kept at 290 °C for 1 h under reflux. After the solution was cooled down to room temperature, 50 mL of ethanol was added into the flask that was left on a strong magnet for 6 h to collect the products. Finally, the products were washed three times with ethanol and dispersed in chloroform at a concentration of 1 mg/mL for later use.

### Synthesis of clustered Fe_3_O_4_ nanoparticles

Step 1: PVP (0.58 g, molecular weight 58,000) was dissolved in 5 mL ethylene glycol under magnetic stirring. Step 2: DTAB or TTAB or DeTAB (0.02 g) were dissolved in 1 mL pure water, which was then combined with 10 mg individual ferric tetroxide (Fe_3_O_4_) nanoparticles in chloroform solution. Step 3: after 3 min vigorous vortex, the mixture in step 2 was transferred into a three-necked flask that was incubated at 40 °C in a water bath, while chloroform was removed in a nitrogen flow (this process takes about 3 min). Step 4: The ethylene glycol solution from step 1 was added into the mixture from step 3 (with a pipette gun, within 15 s) and mechanically stirred for 10 min at a rotation speed of 350 rad·min^−1^. Then, the resulting mixture was then heated to 80 °C and maintained at that temperature for 6 h. After cooling down to room temperature, the as-formed clusters were divided into two 10 mL centrifuge tubes, washed once with ethanol and centrifuged at 5000 rpm for 15 min to collect the final products that were dispersed in ethanol at a concentration of 20 mg/mL. The morphology of the as-synthesized nanoparticle clusters was characterized with a JEOL JEM-2100 transmission electron microscopy (TEM).

### Dynamic light scattering (DLS)

The synthesized Fe_3_O_4_ NPCs were diluted in RPMI 1640 medium to a final concentration of 0.25 mg/mL. The particle size distribution was determined by DLS on a Malvern Nanosizer Zen 3600 instrument (Worcestershire, United Kingdom) equipped with a 633 nm laser using backscatter technology. The temperature was maintained at 25 °C, and the scattering angle was 173° from the incident laser beam. The mean particle diameter was calculated from the quadratic fit of the correlation function. The particle size distribution was measured using the inbuilt DTS (nano) software. Samples were equilibrated for 1 min before measurement. All measurements were performed in triplicates.

### Hyperthermia effect of Fe_3_O_4_ nanoparticle clusters in solution

The Fe_3_O_4_ nanoparticle clusters were diluted to various concentrations in saline (0.0375, 0.0625, 0.0125, or 0.25 mg/mL). One milliliter of each dilution was transferred into a 10 mL centrifuge tube and irradiated with a 808 nm continuous-wave NIR laser (Changchun New Industries Optoelectronics Technology, Changchun, China) at power densities of 0.7, 1, 2, and 4 W·cm^−2^ with a spot size of 5 mm. Pre- and post-irradiation temperatures were recorded by a thermocouple positioned 1 cm beneath the solution surface. Measurements were made every 60 s over a period of 10 min.

### In vitro cytotoxicity assay

A375 melanoma cell line was provided by the Core Laboratory at China–Japan Union Hospital of Jilin University. Use of this cell line was approved by the Animal Experimental Ethical Inspection Committee at Jilin University (Permit no.: 201802034). Cell culture was performed as previously described [15]. After reaching 70–80% confluence, they were seeded into 96-well plates at the density of 10^4^ cells per well and cultured for 24 h, after which they were treated with Fe_3_O_4_ nanoparticle clusters at different concentrations (0, 0.0375, 0.0625, 0.0125, or 0.25 mg/mL) for 4 h in the presence or absence of 808 nm NIR irradiation (power density of 1 W·cm^−2^ for 10 min). After another 24 h, cells were examined by light microscopy and MTT assays were performed as per the manufacturer’s instructions. The Optical absorbance was measured at 490 nm using a microplate reader. Cell viability was calculated as percentage of the OD value of the treatment group relative to that of the control group. All experiments were repeated three times.

### Flow cytometry

Flow cytometry analysis was performed as previously described [15]. Briefly, A375 cells were treated with 0.0625 mg/mL NPCs or vehicle control for 4 h, then exposed to NIR irradiation for 10 min or left intact. After 24 h, cells were washed with PBS, pelleted by centrifugation and underwent Annexin V and propidium iodide staining as per manufacturer’s instructions (ThermoFisher Scientific, Waltham, USA). Samples were analyzed in the FACS Calibur flow cytometry (BD Biosciences, San Jose, USA). At least 10,000 events of single cells per sample were collected.

### Western blot

A375 cells were thawed and seeded in a 6-well plate to equilibrate for 24 h prior to experiments. Then, they were either left intact (as control) or treated for 4 h with Fe_3_O_4_ nanoparticle clusters at concentrations of 0.125 or 0.25 mg/mL. Then, the cells were irradiated with 808 nm NIR laser for 10 min at a power of 1 W. Twenty-four hours after continued culturing, whole cellular protein lysates were prepared by in-well scrapping in RIPA buffer supplemented with protease inhibitors (Roche), followed by centrifugation at 12,000*g* for 10 min at 4 °C to save the supernatant. Protein concentrations were determined by the BCA Protein Assay Kit according to the manufacturer’s instructions (Thermo Fisher). Western blot was performed as previously described with modifications [23]. Briefly, equal amounts of 15 µg total proteins were resolved by SDS-PAGE, transferred to nitrocellulose membranes and blocked with 5% non-fat milk for 1 h at room temperature. After overnight incubation with anti-HSP70 primary antibody (Cell Signaling Technologies, #4872) or anti-ACTB (Cell Signaling Technologies, #4970) at 4 °C, membranes were washed with 1× TBST for three times and incubated for 1 h at room temperature with HRP-conjugated Goat anti-Rabbit secondary antibody (Thermo Scientific). Membranes were washed three times with 1× TBST and rinsed in H_2_O_2_/DAB substrate mixture until the reaction proceeded to the desired intensity. Finally, membranes were washed in water, dried and photographed by ChemiDoc™ MP Imaging System (Bio-Rad). Densitometric quantification analysis was performed using the built-in Image Lab software. Relative expression levels were presented as ratios of band intensities of HSP70 over the internal control gene GAPDH.

### Semi-quantitative reverse transcription-PCR (RT-PCR) and qPCR

Treatments of A375 cells were identical to those mentioned above. Total RNAs were isolated via TRIzol method following the vendor’s manual (Invitrogen) and quantified by NanoDrop. After DNase I treatment (Roche), 1 µg RNA was reverse-transcribed to cDNA using HiScript II 1st Strand cDNA Synthesis Kit (Vazyme). Benchtop PCR was performed on a GeneAmp PCR System 9700 machine in a 50 µL reaction volume: 1× PCR buffer (with Mg^2+^), 0.2 mM dNTP, 0.2 µM primers, 50 ng of template cDNA, and 0.5 µL of Platinum TaqDNA polymerase (Invitrogen). The cycling parameters used were as follows: initial 94 °C for 5 min, followed by 30 cycles of 94 °C for 30 s, 55 °C for 30 s and 72 °C for 60 s, an additional extension at 72 °C for 7 min, and finally hold at 4 °C. The PCR products were resolved by electrophoresis using agarose gels supplemented with ethidium bromide. Images of bands were acquired by ChemiDoc™ MP Imaging System (Bio-Rad) and quantification was performed using the built-in Image Lab software. Relative expression levels were presented as ratios of band intensities of HSP70 over the internal control gene GAPDH. qPCR was performed using Hieff SYBR Green Master Mix (YEASEN, #11203ES03, Shanghai, China) on 7300 Plus Real-Time PCR Instrument (Applied Biosystems, Foster City, CA, USA) according to the manufacturer’s instructions. Primer sequences for *Hsp70* are: 5′- TTTTGGTCCTAAGAATCGTTCA-3′ (forward) and 5′- ACACTTTCGGCTGTCTCCTTCA-3′ (reverse), for *Gapdh* are 5′-GGGTGATGCTGGTGCTGAGTATGT-3′ (forward) and 5′-AAGAATGGGTGTTGCTGTTGAAGTC-3′ (reverse).

### In vivo photothermal therapy

A total number of 2 × 10^6^ cultured A375 cells were resuspended in normal saline and injected subcutaneously at the dorsal side of the right hind leg of BALB/c nude mice (averaged body weight 20 g), which were returned to their cages for four days until tumors grew to at least 5 mm in diameter. Thereafter, these mice were anesthetized with 3% isoflurane and injected intratumorally with Fe_3_O_4_ nanoparticle clusters (2.5 mg/kg body weight) or saline vehicle, then either left undisturbed or irradiated at the tumor site with 808 nm NIR laser for 10 min at 1 W·cm^−2^. This irradiation procedure was repeated four times at 12 h intervals, leading to the following treatment groups (*n* = 8 per group): 1) saline-alone, 2) saline + NIR, 3) nanoparticle clusters-alone, and 4) nanoparticle clusters + NIR. Naïve tumor-free BALB/c nude mice served as normal control. Body weight, tumor volume, and abnormal behaviors were closely monitored during the study.

### Immunohistochemistry

At the end of in the vivo photothermal therapy, mice were euthanized and fixed with 4% paraformaldehyde via intracardiac perfusion. Tumors were then collected, sectioned and analyzed by immunohistochemistry to investigate expression of HSP70 according to a standard protocol [24]. Images were captured using a bright-light microscope (Olympus).

### Statistical analyses

All the data were analyzed by GraphPad Prism 8.0 and expressed as mean ± SD. One-way ANOVA was applied for comparison among multiple groups, followed by post hoc test. Student *t*-test (two-tailed) was used for comparison between two groups. All statistical differences with *p* values below 0.05 were considered significant.

## Supporting Information

File 1 Additional figures.
