# Supplementary material for: Photothermal ablation of murine melanomas by Fe3O4 nanoparticle clusters
Source: Beilstein J Nanotechnol. 2022 Feb 22;13:255–64. doi: 10.3762/bjnano.13.20 (PMC8895026; doi:10.3762/bjnano.13.20)
Supplement: File 1 — Additional figures. [file Beilstein_J_Nanotechnol-13-255-s001.pdf]

BEILSTEIN JOURNAL OF NANOTECHNOLOGY

Supporting Information

for

Photothermal ablation of murine melanomas by Fe_3O_4 nanoparticle clusters

Xue Wang, Lili Xuan and Ying Pan

Beilstein J. Nanotechnol. **2022,** *13,* 255–264. doi:10.3762/bjnano.13.20

Additional figures

License and Terms: This is a supporting information file under the terms of the Creative Commons Attribution License (https://creativecommons.org/licenses/by/4.0). Please note that the reuse, redistribution and reproduction in particular requires that the author(s) and source are credited and that individual graphics may be subject to special legal provisions.
The license is subject to the *Beilstein Journal of Nanotechnology* terms and conditions: (https://www.beilstein-journals.org/bjnano/terms)

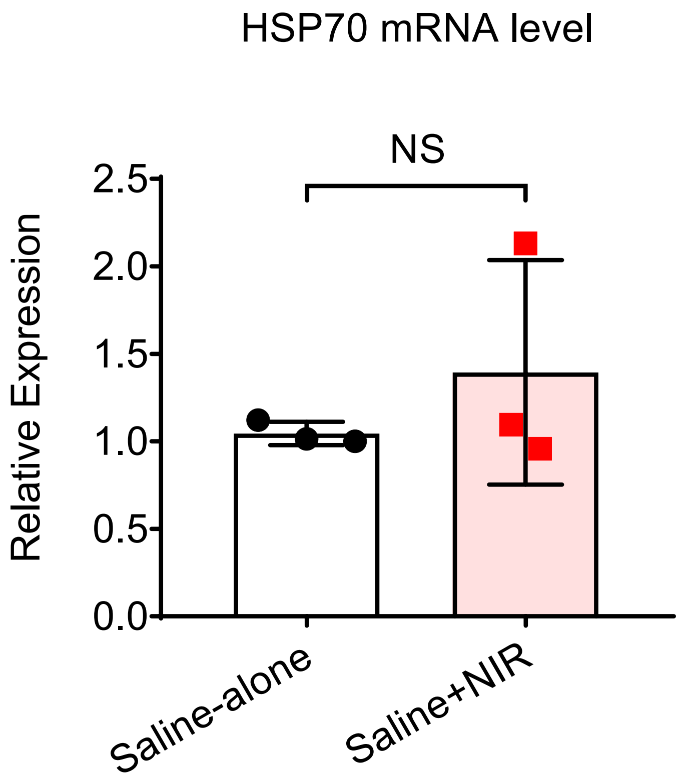

Figure S1: NIR irradiation did not change mRNA expression of HSP70 in A375 cells. A375 cells were treated with vehicle control saline, then left either intact or exposed to NIR irradiation for 10 min. After another 24 h, cells were harvested and qPCR was performed to measure mRNA expression of *Hsp70*. Results were first normalized to the internal standard *Gapdh* and then expressed as fold-change over saline-alone control as determined by the delta-delta CT method. NS, nonsignificant.

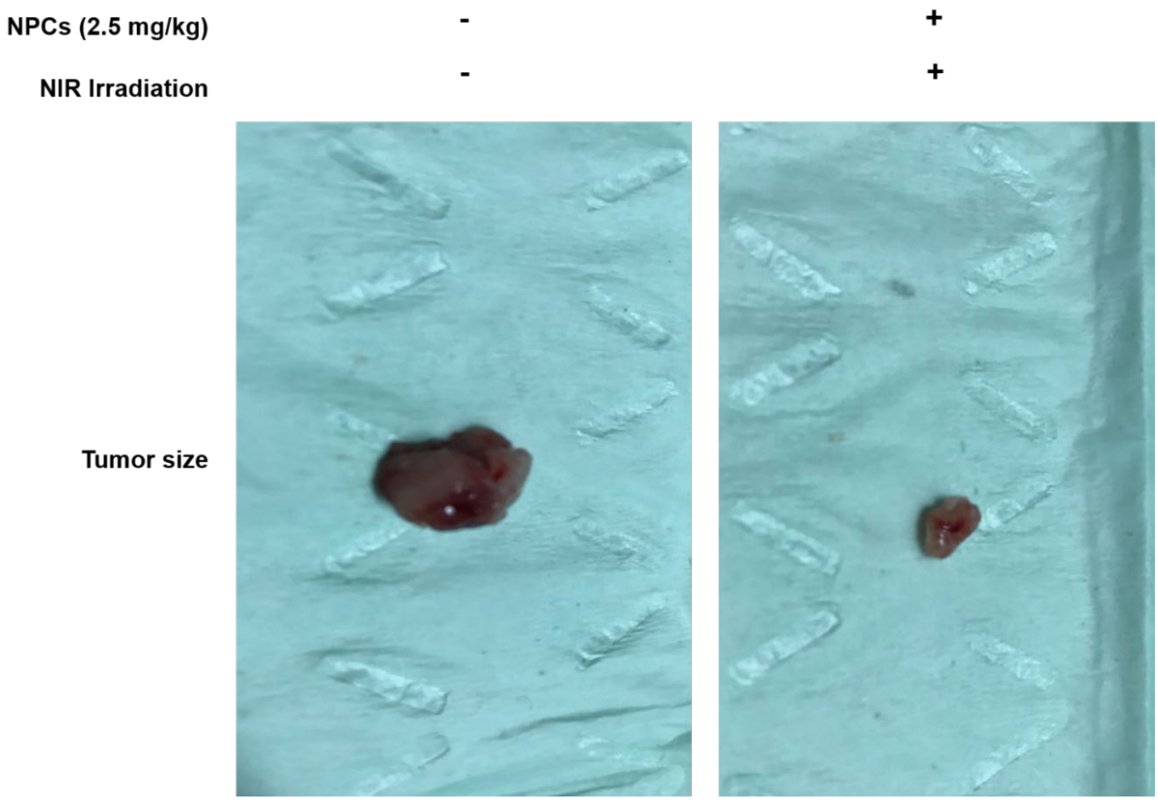

Figure S2: Original full images of harvested tumor xenografts. Tumors were isolated from BALB/c mice that were intratumorally injected with either NPCs or saline, then were irradiated with or without NIR. Pictures were taken using the inbuilt camera of an iPhone. Magnification of the two images is identical as justified by the rectangular marks of similar size present on the background mat.
